# How to Sample Dozens of Substitutions per Site with λ Dynamics

**DOI:** 10.1021/acs.jctc.4c00514

**Published:** 2024-07-08

**Authors:** Ryan L. Hayes, Luis F. Cervantes, Justin Cruz Abad Santos, Amirmasoud Samadi, Jonah Z. Vilseck, Charles L. Brooks

**Affiliations:** † Department of Chemical and Biomolecular Engineering, University of California Irvine, Irvine, California 92697, United States; ‡ Department of Pharmaceutical Sciences, University of California Irvine, Irvine, California 92697, United States; § Department of Medicinal Chemistry, College of Pharmacy, University of Michigan, Ann Arbor, Michigan 48109, United States; ∥ Department of Biochemistry and Molecular Biology, Indiana University School of Medicine, Indianapolis, Indiana 46202, United States; ⊥ Center for Computational Biology and Bioinformatics, Indiana University School of Medicine, Indianapolis, Indiana 46202, United States; # Department of Chemistry, University of Michigan, Ann Arbor, Michigan 48109, United States; ¶ Biophysics Program, University of Michigan, Ann Arbor, Michigan 48109, United States

## Abstract

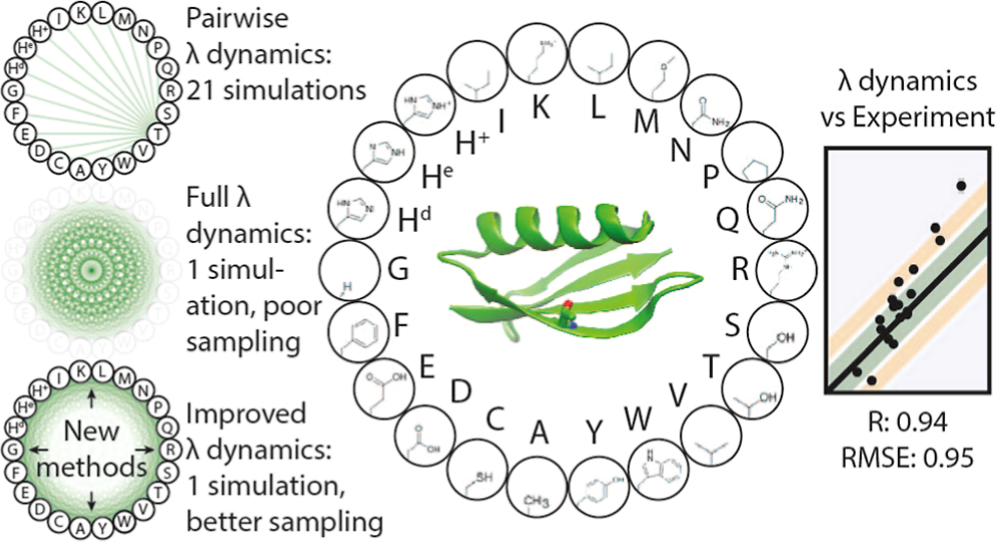

Alchemical free energy methods are useful in computer-aided drug design and computational protein design because they provide rigorous statistical mechanics-based estimates of free energy differences from molecular dynamics simulations. λ dynamics is a free energy method with the ability to characterize combinatorial chemical spaces spanning thousands of related systems within a single simulation, which gives it a distinct advantage over other alchemical free energy methods that are mostly limited to pairwise comparisons. Recently developed methods have improved the scalability of λ dynamics to perturbations at many sites; however, the size of chemical space that can be explored at each individual site has previously been limited to fewer than ten substituents. As the number of substituents increases, the volume of alchemical space corresponding to nonphysical alchemical intermediates grows exponentially relative to the size corresponding to the physical states of interest. Beyond nine substituents, λ dynamics simulations become lost in an alchemical morass of intermediate states. In this work, we introduce new biasing potentials that circumvent excessive sampling of intermediate states by favoring sampling of physical end points relative to alchemical intermediates. Additionally, we present a more scalable adaptive landscape flattening algorithm for these larger alchemical spaces. Finally, we show that this potential enables more efficient sampling in both protein and drug design test systems with up to 24 substituents per site, enabling, for the first time, simultaneous simulation of all 20 amino acids.

## 1 Introduction

**Figure 1:**
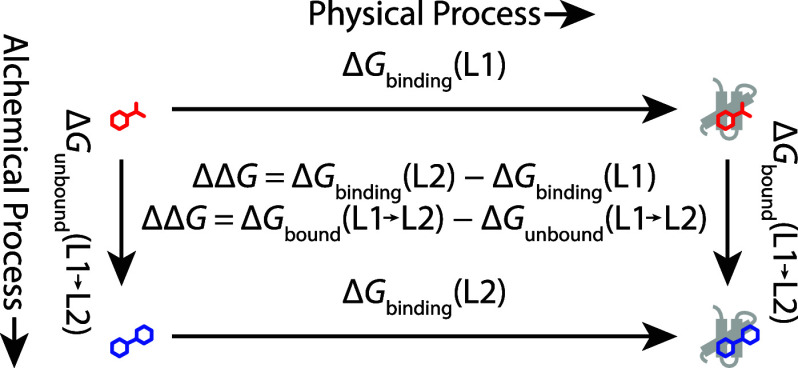
Thermodynamic diagram for computing a relative binding free energy (ΔΔ*G*_bind_). The physical process of binding transfers the ligand from a solvent environment (white) to a protein (gray) environment. The alchemical process converts from ligand 1 (L1 in red) to ligand 2 (L2 in blue). To obtain ΔΔ*G*_bind_, alchemical methods take the difference between the two rapidly converging vertical alchemical processes, rather than the two slowly converging horizontal physical processes. Similar thermodynamic diagrams can be constructed for other free energies of interest (see Supporting Information).

Alchemical free energy methods utilize molecular dynamics simulations to make high quality free energy predictions useful for biophysical insight and biomolecular design. Specific applications include computer-aided drug design,^1−6^ pH-dependent effects,^7−13^ and protein mutation and design.^14−18^ These methods evaluate the relative free energy between related chemical systems for some slowly converging physical process such as ligand binding or protein folding. Such free energy methods are called alchemical because they evaluate free energy differences for the rapidly converging alchemical processes rather than the slowly converging physical processes. Typically they introduce a coupling parameter λ into the potential energy function, such that λ = 0 and λ = 1 correspond to two distinct physical chemical states, and other λ values are nonphysical alchemical intermediates (Figure 1). Many alchemical free energy methods are available including free energy perturbation (FEP),^19^ thermodynamic integration (TI),^20^ the multistate Bennett acceptance ratio (MBAR),^21^ nonequilibrium methods,^22^ enveloping distribution sampling,^23,24^ Gibbs sampling,^25−27^ orthogonal space random walk,^28^ and λ dynamics.^29^

λ dynamics is a particularly scalable and efficient alchemical free energy method that has developed rapidly in recent years. The multisite generalization^30^ enabled exploration of combinatorial chemical spaces within a single simulation that would otherwise require hundreds of FEP or TI simulations.^5,17,31^ The development of implicit constraints focused sampling away from the alchemical intermediates and more onto the physical states of the system.^32^ Both biasing potential replica exchange^33^ and adaptive landscape flattening (ALF)^17,34^ further improved sampling by accelerating transitions between physical states. Adopting soft cores^34^ and particle mesh Ewald (PME) electrostatics^10,17,35,36^ from other methods further improved robustness and accuracy.

The power of λ dynamics lies in the fact that one can generalize from a single dimensional λ variable to a multidimensional alchemical λ space. This enables the characterization of combinatorial chemical spaces for hundreds of systems within a single simulation, in contrast to an infeasible number of pairwise comparisons that would be needed with conventional alchemical free energy methods. As a result, speedups of 1 to 2 orders of magnitude have been observed in various systems.^2,5,30,31^ Previous studies have explored hundreds of drug molecules^5,31^ or hundreds to thousands of protein sequences^17,37^ but have been limited to sampling a maximum of 8 or 9 substituents per site. This limit arises because the volume of phase space corresponding to alchemical intermediates grows exponentially with the number of substituents, and beyond this cutoff, the implicit constraints are less effective at focusing sampling on the physical end states. In prior studies examining more perturbations per site, including a D3R grand challenge^38^ and a commercial λ dynamics benchmark study,^5^ substituents had to be divided into multiple groups sampled in separate simulations to obtain adequate sampling of all physical end states. For a single site, this is not a significant problem, but in protein design problems where one may wish to sample all 20 amino acids at several sites, the number of λ dynamics simulations would rapidly grow.

In this work, we address this limitation by introducing new biasing potentials that refocus sampling on the physical end states of the system and a more scalable ALF algorithm that flattens alchemical barriers in these higher dimensional spaces. Two biasing potentials on the implicit constraint variable θ are presented. The second bias scales well to 1000 substituents or more. Next, we note that the current ALF algorithm, which makes a linear approximation leading to a quadratic loss function, scales as *N*_*s*_^4^, where *N*_*s*_ is the number of substituents. Implementing a full nonlinear loss function improves the scaling to *N*_*s*_^2^ and allows ALF to converge with fewer cycles of molecular dynamics sampling. Adding a small likelihood term to the loss function further improves convergence. These developments are tested on solvation free energies of 1,4-substituted benzene derivatives, on folding free energies for sampling all 20 amino acid mutations within protein G, and with the protein receptor p38 and the calculation of small molecule binding free energies with 16 and 12 substituents sampled at two sites. Together, these developments increase the limit on the number of substituents that can be practically sampled with λ dynamics to between 40 and 100 per site.

## 2 Theoretical Methods

### 2.1 λ Dynamics

The λ dynamics potential energy for a system, (*U*), is given by
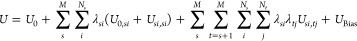
1where *U*_0_, *U*_0,*si*_, *U*_*si*,*si*_, and *U*_*si*,*tj*_ are the interactions within the environment, between alchemical groups and the environment, within an alchemical group, and between different alchemical groups, respectively. These interaction terms may also be functions of λ for soft-core interactions,^34^ soft bonds,^39^ or PME electrostatics.^10,35,36^*U*_Bias_ is a biasing potential on the set of λ variables to optimize sampling by flattening alchemical barriers, *M* is the number of sites, and *N*_*s*_ is the number of substituents at site *s*. In addition, the constraints
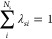
2
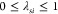
3are imposed implicitly though transformation to the variables θ_*i*_^32^
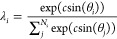
4where *c* = 5.5 is typically chosen, and the θ variables are allowed to propagate according to the forces – ∂*U*/∂θ exerted by the potential energy function in a molecular dynamics simulation.

In order to obtain free energies, λ values near the alchemical end points are binned, and the relative populations are used to estimate relative free energies.
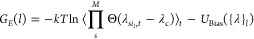
5where *G*_*E*_(*l*) is the free energy of ligand (or sequence) *l* in the ensemble *E*,  is the λ value of the substituent *i*_*l*_ of ligand *l* at site *s* at time *t*, angle brackets denote the ensemble or trajectory average over time, λ_c_ is a cutoff of typically 0.99 that defines when a substituent is in an approximately physical state rather than an alchemical intermediate state, Θ(*x*) is the Heaviside function (a boolean indicator whether *x* > 0), the product over *s* ensures a frame is only counted if  is above λ_c_ at all *M* sites, and *U*_Bias_({λ}_*l*_) is the value of the bias potential for ligand *l*. Alchemical free energy differences for a particular ensemble are given by
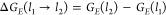
6and the difference in Δ*G*_*E*_(*l*_1_ → *l*_2_) between two different ensembles gives the ΔΔ*G* of interest.

Other approximate estimators are available for systems with multiple sites, including the independent site estimator
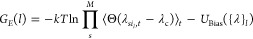
7that neglects coupling between sites^5^ or the Potts estimator that only includes pairwise couplings.^40^ Unbiased estimators that do not depend on λ_c_ are also available,^21,25−27^ but are prohibitively expensive for continuous λ dynamics simulations in large alchemical spaces (see Supporting Information).

### 2.2 Bias on θ to Focus Sampling on End Points

In eq 5, states for which  for all sites are considered approximately physical and represent the fraction of time that one ligand is sampled. Summing over all ligands gives the fraction physical ligand (FPL).
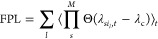
8

Conversely, 1 – FPL corresponds to the fraction of the time the simulation samples nonphysical alchemical intermediates. The time sampling alchemical intermediates does not contribute to the free energy estimates (eq 5) and is largely wasted, but it should not be eliminated entirely because it is required to maintain high transition rates between physical states that promote converged free energy estimates. For optimal sampling, the FPL should be high and evenly distributed between ligands because if  is too low for a ligand, estimates of *G*_*E*_(*l*) for that ligand may be noisy. In practice, values of at least 0.01 are usually sufficient, but values above 0.5 may indicate low transition rates or uneven sampling.

**Figure 2:**
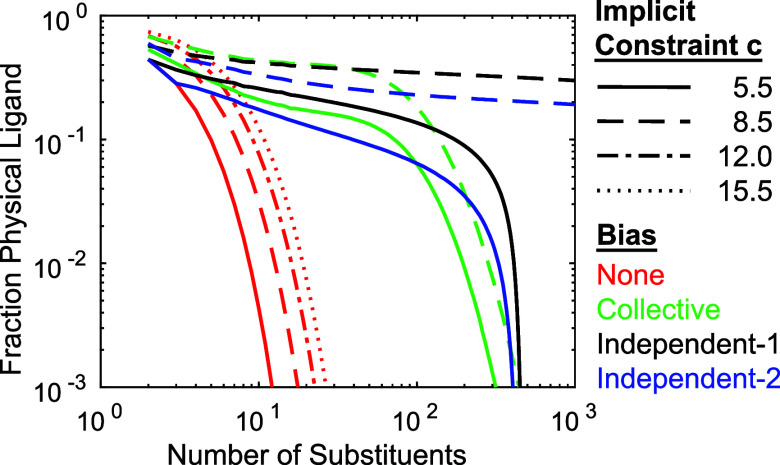
With no bias (red curve) and the standard implicit constraint value of *c* = 5.5 (solid line), the FPL with λ > 0.99 falls to 0.01 at just 9 substituents. Increasing *c* only helps a little. The collective bias on θ (green) preserves a much higher FPL, but begins to fail between 100 and 200 substituents. The independent biases on θ (black and blue) are much more effective. The standard *c* value of 5.5 fails between 300 and 400 substituents because even when one λ value is clearly dominant, it cannot reach 0.99 because of the many terms in the denominator of eq 4. Increasing *c* to 8.5 (dashed line) suppresses these terms and allows the independent bias to maintain high FPL beyond 1000 substituents.

For a single site with two or three substituents, the FPL is high on a flat alchemical landscape^34^ (0.44 and 0.28, respectively), but at 9 substituents, the FPL falls below 0.01, and it continues to fall by a factor of 2 for each additional substituent added thereafter (Figure 2). The problem is exacerbated if multiple sites are perturbed concurrently. For independent, noninteracting sites, the full FPL is the product of the individual site FPL values.

Several strategies exist for increasing the FPL. A single λ value will be greater than the λ_c_ = 0.99 cutoff when a single term in the sum in the denominator of eq 4 is sufficiently dominant. This occurs when one θ value is near π/2 and the remaining θ values are near – π/2. Since each substituent has its own value of θ, the probability of only having one θ value near π/2 becomes vanishingly small for large numbers of substituents. Increasing the constant *c* in the implicit constraints is one way to raise FPL, but large increases compromise numerical stability, so modest increases to no more than 15.5 are preferred,^31,40^ but these only stave off low FPL for a few extra substituents (Figure 2). An alternative strategy is to add biasing potentials to penalize alchemical intermediates. In previous work, small barriers were placed between the alchemical end points to decrease the population of alchemical intermediates; however, these barriers must remain small or they will slow transition rates and convergence of the alchemical simulations.^31,40^ For large numbers of substituents, these small barriers were insufficient. We briefly considered new biases on λ (see Supporting Information) but found biases on θ more effective.

In order for a bias on θ to maintain high FPL, it must favor states with a single θ value near π/2 and all remaining θ values near – π/2. The following collective bias on θ does this by counting the θ values near π/2 as *n*_+_ and the θ values near – π/2 as *n*_–_
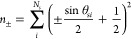
9*n*_+_ and *n*_–_ are then harmonically restrained to their desired values of 1 and *N*_*s*_ – 1.
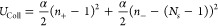
10

With a modest coefficient of α = *kT*, this bias maintains high values of FPL between 0.16 and 0.21 for 10 to 30 substituents (Figure 2). For hundreds of substituents, the FPL falls precipitously by about a factor of 10 per hundred substituents, though this decay can be slowed with a higher *c* constant in the implicit constraints (Figure 2).

An alternative independent bias is comparably effective for dozens of substituents and more effective for hundreds of substituents. In this case, a bias is added for every single θ coordinate
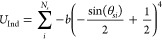
11

The bias coefficient *b* is calibrated to ensure high FPL. Calibrating the bias so that on average one θ coordinate is between 0 and π, and the remaining θ coordinates are in the trap between π and 2π (independent-1) lowers transition rates below the rates observed with the collective bias (see Supporting Information eq S7). Instead, the bias is calibrated so that on average two θ coordinates are between 0 and π (independent-2), which gives comparable transition rates to the collective bias (Table 1). The independent-2 coefficient is given by
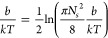
12which is solved iteratively for *b*/*kT* (see Supporting Information for derivation). For two and three substituents, no solution exists, and *b* = 0 is used to turn off the bias.

**Table 1:** Transition Rates Reveal Improved Sampling for Many Substituent Systems with New θ Biases^a^

	1,4-benzene system	
θ bias	8 × 8	24 × 24
none	73.4 ± 0.5	14.3 ± 0.1
collective	120.5 ± 0.8	114.3 ± 0.6
independent-1	87.8 ± 0.5	99.8 ± 0.4
independent-2	113.1 ± 0.8	121.4 ± 0.5

a Transition rates per ns during 20 ns production runs of 1,4-substituted benzene derivatives in the solvated ensemble after landscape flattening. Results are averaged over both sites in 5 trials with 2 (8 × 8) or 5 (24 × 24) replicas each. Uncertainties are the standard error of the mean.

The independent bias (independent-2) using the coefficients in eq 12 has slightly lower FPL than the collective bias, but the comparable transition rates and superior scaling to hundreds of substituents make it a better choice. If higher FPL is needed, it can be achieved by raising the implicit constraint *c* parameter. Supporting Information Figure S1 shows the significant improvement in sampling given by the independent-2 bias. There are still some applications where the collective bias may be desirable, for example, when trying to significantly increase the FPL of just a few substituents in constant pH or semigrand canonical sampling where nonphysical intermediate states should be avoided. Consequently, the independent bias (independent-2) with the coefficients in eq 12 is used for the remainder of this study.

### 2.3 Nonlinear Adaptive Landscape Flattening with Improved Scaling

With the independent bias, one can in principle characterize arbitrarily large numbers of substituents at a single site; however, one immediately runs into limitations in the adaptive landscape flattening (ALF) algorithm. The bias on θ to increase FPL is added to eq 1 along with the existing bias *U*_Bias_ that is tuned by ALF to flatten alchemical barriers. Because the system freely diffuses through alchemical space, large free energy barriers or traps can slow convergence beyond accessible time scales and must be flattened to optimize sampling. ALF runs many cycles. Each cycle consists of a very short simulation followed by calculation of *N*_*s*_^2^ free energy profiles and estimation of changes in *N*_*s*_ + 5*N*_*s*_(*N*_*s*_ – 1)/2 bias parameters that lower barriers in those free energy profiles (see Supporting Information for a description of profiles and bias potentials). The current linearized ALF algorithm performs well for 9 substituents, but its computational cost scales like *N*_*s*_^4^, so it quickly becomes rate limiting for larger numbers of substituents. For the folded ensemble of the 22 substituent protein G test system described later, molecular dynamics takes 23 000 s on an NVidia A30 GPU, but bias parameter optimization takes over five times longer (Table 2). Consequently, we introduce a new nonlinear ALF algorithm that scales like *N*_*s*_^2^, which reduces the time for bias parameter optimization (Table 2). This improved ALF algorithm becomes rate limiting between 30 and 40 substituents and should allow sampling of 50 to 100 substituents before ALF becomes impractical. While this is a significant improvement over the previous ALF algorithm, it is still significantly less than the 1000 substituents enabled by the bias on θ; thus, ALF determines the limit in the number of substituents that can be sampled.

**Table 2:** Timing 330 Cycles of ALF Loss Function Optimization in Protein G with 22 Substituents on an NVidia A30 GPU Shows New Nonlinear ALF Scales Better than Linearized ALF

ALF	Bins	*P*_ln *L*_	time (s)
linearized	400	no	124 311
nonlinear	64	no	11 818
nonlinear	64	yes	11 400
nonlinear	256	no	9 541
nonlinear	256	yes	10 306

The loss function for nonlinear ALF is
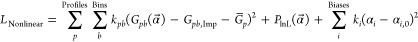
13

**Figure 3:**
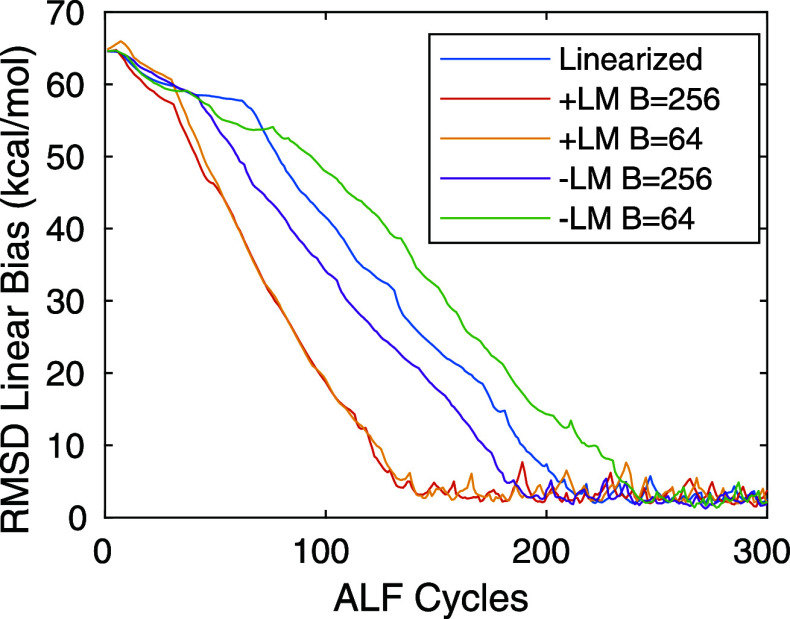
Convergence of the linear bias parameters as a function of ALF cycles for the folded ensemble of the 22 substituent Protein G test system. Convergence is quantified by the root-mean-square difference (RMSD) of all bias parameters relative to final biases obtained from an independent ALF run comprising 5 × 100 ns production runs, using nonlinear ALF with likelihood optimization and *B* = 256. Nonlinear ALF with likelihood optimization (+LM) for 256 bins provides the fastest convergence.

In the first term, the sum on *p* runs over all 1D, transition, 2D, and intersite 2D profiles, as described previously,^17,40^ and the sum on *b* runs over all bins of each free energy profile that were sampled. Both *B* = 64 and *B* = 256 bins were considered, but *B* = 256 typically converges to the optimal biases in fewer cycles of sampling (Figures 3 & S2). The coefficient *k*_*pb*_ penalizes deviations from flat profiles and is scaled to eliminate *B* dependence,  is the free energy of profile *p* and bin *b*, reweighted to bias parameters α⃗ by WHAM/MBAR^41^ (see Supporting Information), *G*_*pb*,Imp_ is the intrinsic free energy of the implicit constraints when sampling a flat landscape, determined by Monte Carlo sampling, and  is the weighted average of the free energy profile after subtracting *G*_*pb*,Imp_. The second term  is the negative log likelihood, as described below. In the third regularization term, α_*i*_ is biasing potential parameter *i*, and *k*_*i*_ and α_*i*,0_ are regularization terms to prevent excessive changes in α_*i*_. Following the approach in ref (42), we minimize the loss function with a limited memory Broyden–Fletcher–Goldfarb–Shanno algorithm. We halt the minimization after the root-mean-square change in the biasing coefficients is lower than 1.25 × 10^–3^ kcal/mol two steps in a row (see Supporting Information).

Linearized ALF employs a similar loss function, but without the log likelihood term, with *B* = 400, and with the approximation
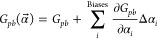
14where *G*_*pb*_ is the free energy with the current bias parameters and Δα_*i*_ is the proposed change in bias coefficient α_*i*_. Nonlinear ALF scales like *N*_*s*_^4^ because there are *O*(*N*_*s*_^4^) derivatives that must be computed.

The log likelihood optimization term finds the most likely bias away from a flat free energy landscape to have produced the observed distribution of states. Likelihood optimization is an intuitive way to perform ALF but fails badly when applied alone. Likelihood optimization is helpful for driving flattening of trapped degrees of freedom early in flattening when most bins of the corresponding profile are unsampled and the first term of eq 13 only provides a weak drive to improve the bias. Consequently, adding likelihood optimization with a small coefficient significantly improves the convergence of ALF (Figures 3 & S2). The negative log likelihood is given by
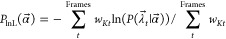
15where *w*_*Kt*_ is the weight of each frame of the trajectory determined by WHAM (see Supporting Information),  is the state of the alchemical variables in that frame, and α⃗ is the proposed value of the biasing potentials. The log probability of observing  given α⃗ is then
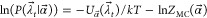
16where  is the energy of the alchemical biases, and *Z*_MC_ is an estimate of the partition function
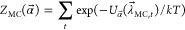
17

The partition function is estimated using a Monte Carlo sampled trajectory  with the same number of frames as the actual trajectory. This trajectory is obtained by sampling the θ variables subject to the biases described above to calculate the λ variables. This trajectory is also used to compute *G*_*pb*,Imp_ values instead of using pretabulated values as in the linearized case.

While the linear bias coefficients must converge to the same values every time ALF is run (Figure 3), other coefficients do not converge to unique values (see Supporting Information Figure S2), and end point biases can differ by 5 to 7 kcal/mol between runs. This variation occurs in both linearized and nonlinear ALF and is undesirable as it blocks a few transition paths with very large barriers to make the remaining profiles flatter. This variation did not occur as extensively for smaller numbers of substituents and suggests that the existing bias potentials do not fit higher dimensional alchemical landscapes as well.

## 3 Test Systems

Three systems are examined to test the θ bias and nonlinear ALF methods described above. We begin with solvation free energies for a previously described symmetric set of 1,4-substituted benzene derivatives.^26^ These solvation free energies converge rapidly and include many copies of the same ligand, so they provide an initial test for correctness. Next, we look at folding free energies of all point mutations to T16 in protein G^43^ and compare them against pairwise calculations and experiment. Finally, we look at a complex set of free energy calculations for ligand binding to the protein receptor p38 with two sites of substitution on the ligand. We compare these to calculations carried out in smaller sets as well as the experimental findings.^44^

### 3.1 Simulation Methods

Simulations were run using the BLaDE module^45^ within the CHARMM molecular dynamics package.^46,47^ Simulations utilized the TIP3P force field for water,^48^ the CGENFF force field for small molecules^49^ (parametrized with MATCH^50^ or paramchem^51,52^), and the CHARMM36 force field for proteins.^53^ Force switching was used for van der Waals interactions with a switching radius of 9 Å and a cutoff radius of 10 Å.^54^ PME electrostatics were used for long-range electrostatics by scaling charges by λ, as described previously,^10,35,36^ using a cutoff of 10 Å, an interpolation order of 6, κ = 0.32 Å^–1^, and a grid spacing of approximately 1.0 Å. Simulations were run with a time step of 2 fs, a Langevin thermostat, and a Monte Carlo barostat.^45^ Ligand perturbations were set up with msld-py-prep.^55^ Protein perturbations were set up with the recently developed whole residue perturbation strategy.^39^ Bonds, angles, and improper torsions are unscaled by λ, while dihedrals and CMAP terms^56^ are scaled by λ. Uncertainties in the free energy are reported as the standard deviation from bootstrapping over the 5 independent trials. Uncertainties in error metrics are reported as symmetric 95% confidence intervals from bootstrapping over different ligands or sequences (see Supporting Information).

### 3.2 1,4 Benzene Derivatives

**Figure 4:**
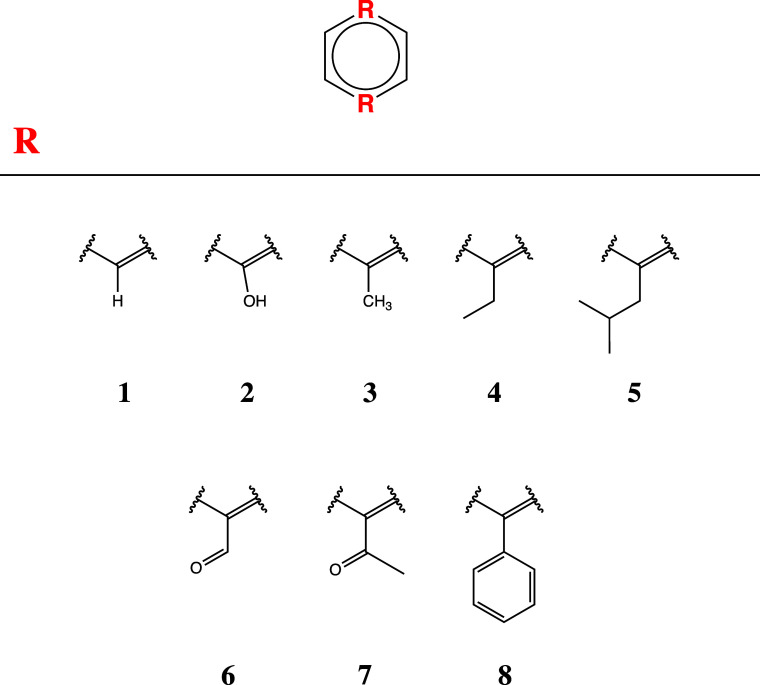
Perturbations considered in the 1,4-substituted benzene system. All 8 perturbations are considered at both sites.

We chose to evaluate solvation free energies for a previously studied set of 1,4-disubstituted benzene derivatives^26^ as an initial test of our methodology (Figure 4). We include 8 identical substituents at both sites for an 8 × 8 system, giving two copies of each differently substituted molecule and one copy of each identically substituted molecule. This system is near the edge of what can be sampled with current methods and allowed comparison of transition rates for different biases, as described previously in Table 1. Furthermore, we studied another system with three copies of each substituent at each site giving a 24 × 24 system, with 18 copies of each differently substituted molecule and 9 copies of each identically substituted molecule.

The system was parametrized with MATCH^50^ because MATCH uses bond charge increment rules for charge assignment, and this provides more localized perturbations to charges, which is necessary due to the close proximity of the alchemical regions. CATS restraints^39^ were added to the benzene carbon in each substituent to allow scaling of double counted angles from that carbon to the core hydrogens; otherwise, the double counted interactions increase hydrogen oscillation frequency beyond the point of numerical stability. Ligands were placed in a cubic box with edges of approximately 35 Å. No ions were included, and periodic boundary conditions were employed for simulations with or without water. Multiple copies of the system are simulated in parallel to provide the increased sampling required to explore the increased number *N*_*s*_(*N*_*s*_ – 1)/2 of transition paths during flattening. ALF scripts allow Hamiltonian replica exchange, so multiple copies of the system were implemented as trivial replica exchange with identical Hamiltonians to allow minimal modification of ALF scripts. Simulations used 2 replicas for the 8 × 8 system and 5 replicas for the 24 × 24 system. Flattening was run for 200 cycles of 100 ps, followed by 20 cycles of 1 ns. Production utilized 5 independent trials to assess statistical reproducibility and included 5 ns to further improve biasing potentials followed by 20 ns simulations for final production. This is significantly more sampling than is required to merely determine relative free energies and further serves to decrease statistical noise to expose systematic errors.

**Figure 5:**
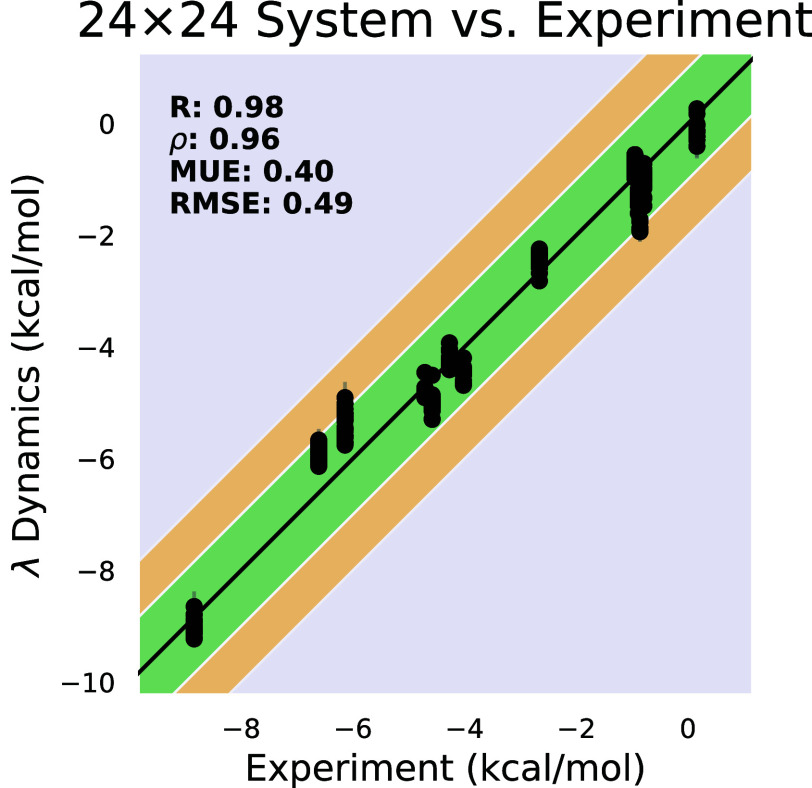
Correlation between λ dynamics calculations and experimental results for solvation free energies of 1,4-substituted benzene derivatives in the 24 × 24 system setup. RMSE and Pearson correlation values of 0.493 ± 0.038 kcal/mol and 0.985 ± 0.003 are obtained. Experimental data taken from refs (57, 58). The regions between ±1 kcal/mol and ±2 kcal/mol are shaded in green and orange, respectively. The solid black line is *y* = *x*.

The centered root-mean-square error (RMSE)  between free energy estimates for identical molecules was evaluated to assess convergence. The centered RMSE is preferred over the raw RMSE  because the raw RMSE is more susceptible to errors in the arbitrary zero point of the relative free energy estimates, usually a reference compound or the native sequence. Comparisons were made between identical molecules within the 8 × 8 system, within the 24 × 24 system, and between the 8 × 8 and 24 × 24 systems for the vacuum ensemble, the solvated ensemble, and the solvation free energy (Table 3). These RMSE values are small and consistent with uncorrelated Gaussian noise as well as with computational uncertainty (see Supporting Information Table S1). Furthermore, the results of λ dynamics calculations were compared to systems for which experimental results^57,58^ are known (Figures 5 & S3). RMSE values of 0.450 ± 0.102 and 0.493 ± 0.038 kcal/mol and Pearson correlation values of 0.987 ± 0.007 and 0.985 ± 0.003 were obtained for the 8 × 8 and 24 × 24 systems, respectively.

**Table 3:** Computational Consistency of 1,4-Substituted Benzene Solvation Free Energies Assessed by RMS Differences in Free Energy between Distinct Identical Molecules (kcal/mol)

	vacuum	solvent	solvation
	Δ*G*	Δ*G*	ΔΔ*G*
8 × 8 vs 8 × 8	0.053 ± 0.009	0.113 ± 0.023	0.139 ± 0.027
24 × 24 vs 24 × 24	0.118 ± 0.003	0.180 ± 0.004	0.215 ± 0.005
8 × 8 vs 24 × 24	0.094 ± 0.004	0.155 ± 0.007	0.178 ± 0.008

### 3.3 Amino Acid Mutations in Protein G

Next we demonstrate the ability of these methods to sample all 20 amino acids at a single site. We chose protein G as a test system because of a recent experimental study exploring all possible mutations except C and W at all sites on the protein.^43^ After eliminating sites with mutation effects beyond experimental sensitivity, we selected T16 because it had the largest standard deviation of mutation effects. T16 is not a buried site that will be excessively difficult to sample, but it is also not a trivial surface site where mutations have a negligible effect.

Simulations considered all 20 possible amino acids, including 3 possible protonation states for histidine, for a total of 22 substituents. Folded and unfolded simulations of all 22 substituents were run using nonlinear ALF; in addition, 21 pairwise perturbations from threonine to each other substituent were run with linearized ALF as controls. Folded simulations were run starting from the PDB structure 1PGA^59^ at pH 7 with 100 mM NaCl and a 55 Å box providing 10 Å on all sides. At pH 7, all residues were predicted by PropKa to have default protonation.^60^ The unfolded ensemble was simulated with a pentapeptide from residues 14 to 18 in a 40 Å box under the same solution conditions. Pairwise unfolded simulations used a cubic box set up with CHARMM-GUI.^61^ All other simulations used a rhombic dodecahedron box with 29% less volume than a cubic box set up using in house python scripts. Previous studies^17,40^ have observed that PME electrostatics give marginally improved results over force switching (fswitch) electrostatics,^54^ especially for longer simulations; we test both approaches for completeness, although PME is clearly more widely used. Several mutations include charge changes, and correction terms for charge changes with PME electrostatics have been developed.^62^ For neutral boxes, the discrete solvent correction is the dominant term, so we apply it to PME calculations, following previous work.^10,37,40^

**Figure 6:**
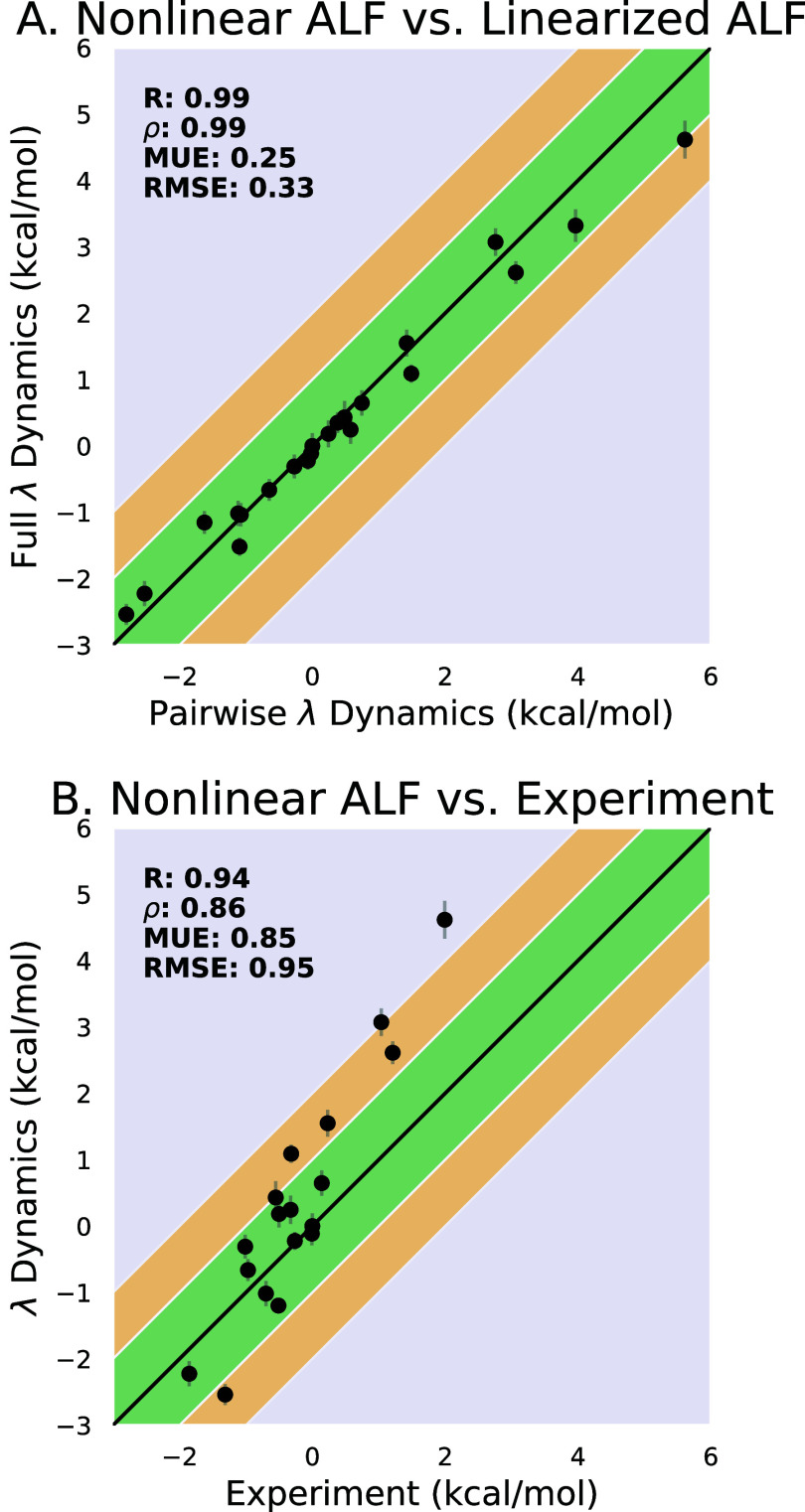
(A) 21 pairwise simulations (*x*-axis) and a single 22 substituent simulation (*y*-axis) run with PME electrostatics show excellent agreement in predicted folding free energies. (B) The single 22 substituent simulation also agrees quite well with the 18 experimental measurements with an RMSE of 0.946 ± 0.289 kcal/mol and a Pearson correlation of 0.937 ± 0.086. The experimental data are taken from ref (43). The regions between ±1 and ±2 kcal/mol are shaded in green and orange, respectively. The solid black line is *y* = *x*.

Comparing results obtained from 21 pairwise simulations run without θ biases to results obtained from the full 22 substituent simulations with θ biases reveals very close agreement (Figures 6A & S4A & Table 4). The centered root mean squared differences are quite small for Δ*G* in both the folded and unfolded ensembles and for the stability ΔΔ*G*_fold_.

**Table 4:** Consistency between Pairwise and 22 Substituent λ Dynamics Assessed by RMS Differences (kcal/mol)

	unfolded	folded	stability
	Δ*G*	Δ*G*	ΔΔ*G*
pme	0.21 ± 0.07	0.26 ± 0.15	0.33 ± 0.11
fswitch	0.15 ± 0.04	0.24 ± 0.09	0.20 ± 0.04

To compare the results of λ dynamics simulations with experimental data, we must combine the results from δ-, ε-, and di-protonated histidine into a single ΔΔ*G*_fold_ value. These three protonation states are denoted as HSD, HSE, and HSP, respectively. One may estimate the Δ*G* of the folded and unfolded states using
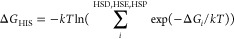
18and take their difference to determine ΔΔ*G*_fold_. Unfortunately, raw Δ*G*_i_^*^ values from simulation include an additive constant γ_*i*_ that is independent of the ensemble and depends only on force field and perturbation pathway.
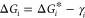
19This constant is typically forgotten because it cancels out between the two ensembles when computing ΔΔ*G*_fold,*i*_, but it must be determined to accurately compute Δ*G*_HIS_ and ΔΔ*G*_fold,HIS_ from eq 18.

To obtain the actual Δ*G*_*i*_ values for use in eq 18 from the raw Δ*G*_i_^*^ values, γ_*i*_ must be determined by running a simulation in a reference ensemble in which Δ*G*_ref,*i*_ is known. For a reference system of histidine with neutral caps on the N and C termini, the p*K*_a_ is 6.53 for protonation at the δ nitrogen (HSE-HSP) and is 6.92 for protonation at the ε nitrogen (HSD-HSP).^63^ We assume a pH of 7, since ref (43) does not indicate the experimental pH. Then, Δ*G*_ref,*i*_ can be computed from p*K*_a_ and pH
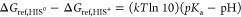
20as −0.109, −0.641, and 0.000 kcal/mol for HSD, HSE, and HSP relative to HSP in the reference system at a pH of 7. Rather than simulate the true reference ensemble, we note that the capped histidine reference and the unfolded ensemble have similar solvation and lack of nearby monopoles, so we approximate the raw reference free energy Δ*G*_ref,i_^*^ by the raw unfolded free energy Δ*G*_unfolded,i_^*^. The correction is then
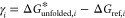
21which is subtracted from the raw results Δ*G*_i_^*^ for each ensemble. The predicted ΔΔ*G*_fold,HIS_ depends on the experimental pH due to the pH dependence of eq 20 (see Supporting Information Figure S5) but remains within 0.6 kcal/mol of the value for a pH of 7 between pH values of 6 and 8.

With a prediction for histidine, we may compare simulation results with experimental values (Figures 6B & S4B & Table 5). Both electrostatic truncation methods give excellent agreement with experiment, but both overpredict the magnitude of mutational effects because the slope of best fit (1.81 for PME and 2.07 for fswitch) is greater than unity. This effect has been observed previously in T4 lysozyme,^17^ where overprediction was also worse for fswitch than for PME electrostatics. Here, PME has a better RMSE, while fswitch has a marginally better Pearson correlation, but the Pearson correlation is less relevant because it does not penalize overprediction. While the 95% confidence intervals in Table 5 from bootstrapping over mutations are large, the RMSE of PME is better than the RMSE of fswitch in 91% (linearized ALF) or 98% (nonlinear ALF) of bootstrap samples. Consequently, this confirms previous observations that PME gives better results for protein mutations.^17,40^ Furthermore, the RMSE of nonlinear ALF is better than the RMSE of linearized ALF in 100% (fswitch) and 93% (PME) of bootstrap samples.

**Table 5:** Comparison of λ Dynamics Results with Experiment Suggests that PME Electrostatics Provide Superior Free Energy Estimates

	linearized ALF	nonlinear ALF
**PME**		
RMSE	1.124 ± 0.427	0.946 ± 0.289
R	0.940 ± 0.092	0.937 ± 0.086
**fswitch**		
RMSE	1.283 ± 0.414	1.165 ± 0.376
R	0.943 ± 0.066	0.943 ± 0.064

### 3.4 Ligand Binding to p38

**Figure 7:**
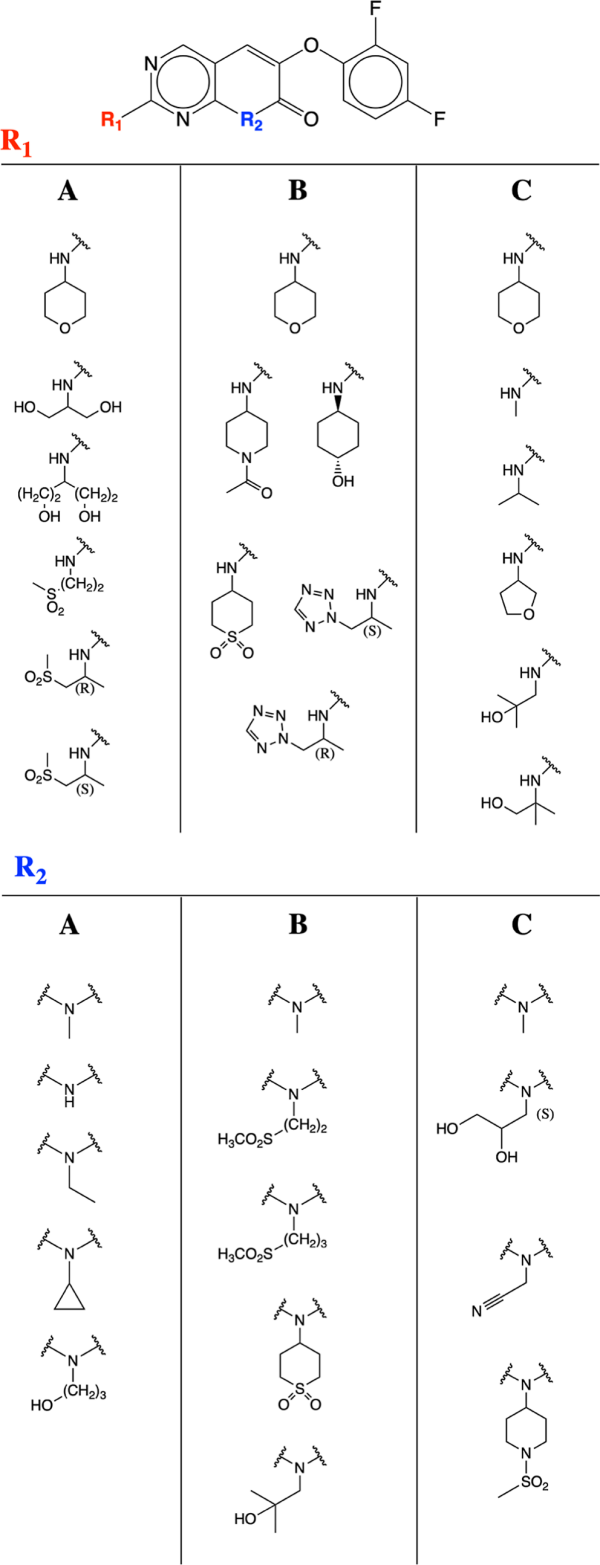
Fragments at each site of variation for the p38 ligand set were subsetted into 3 different groups for both sites 1 and 2. The common core output from msld-py-prep is shown at the top. A total of 9 sets of simulations for each combination of a subset (groups A–C) from site 1 and site 2 were performed using linearized ALF.

We further demonstrate the utility of these methods by calculating the free energy differences for small molecule ligands binding to a protein receptor, p38. This system has been thoroughly tested via multiple free energy calculation protocols and methods.^1,5^ Twenty-seven (27) ligands with experimental binding affinities were selected as the basis for multisite λ dynamics simulations. The 27 ligands were parametrized using ParamChem,^51,52^ and a multiple topology model was generated using msld-py-prep.^55^ The multiple topology model consists of 2 sites with 16 substituents at one site and 12 at another–a 16 × 12 system of a combinatorial set of 192 ligands (Figure 7). Because this system is too large for convergence with linearized ALF, the ligands were divided into nine subsets (see Supporting Information Table S2) sharing the first substituent at each site as a common reference across subsets. The nonlinear ALF simulations included all fragments from both sites. Ligand free energies were computed assuming that the sites are independent, and their free energies are additive, using the independent site estimator in eq 7, originally described as additive estimates by Raman and co-workers.^5^ The multiple topology model included charge renormalization, so the computed relative binding free energies were corrected using a bookending protocol described in ref (55).

A 38.50 Å box providing a 10 Å buffer on all sides was used for the ligand-in-water simulations. The bound simulations were run starting from the PDB ID 3FLZ(44) at pH 7.4 with 100 mM NaCl and a 92 Å box, again providing 10 Å on all sides. Both simulation boxes for p38 were set up via CHARMM-GUI.^61^ The protocols for both linearized and nonlinear ALF were kept as similar as possible for comparison purposes.

The ligand-in-water and bound ligand simulations were run using the ALF schedules described in Tables S3 and S4, respectively. To ensure convergence, final production simulations were extended until an uncertainty of 0.2 kcal/mol or less and of 0.5 kcal/mol was observed for each calculated free energy for the ligands in water and protein environments, respectively. This ensured that the final relative binding free energy value had an uncertainty of roughly 0.5 kcal/mol or less.

Comparing the single ensemble λ dynamics results obtained in either ligand-in-water or bound ligand simulations from both ALF protocols reveals that nonlinear ALF results are very close to those of linearized ALF, as shown in Figure S6. The Pearson R and Spearman coefficient ρ are 1 regardless of the ligand environment being simulated due to the wide range of the data. Likewise, the MUE and RMSE values fall within less than 0.5 and 0.6 kcal/mol, respectively.

**Figure 8:**
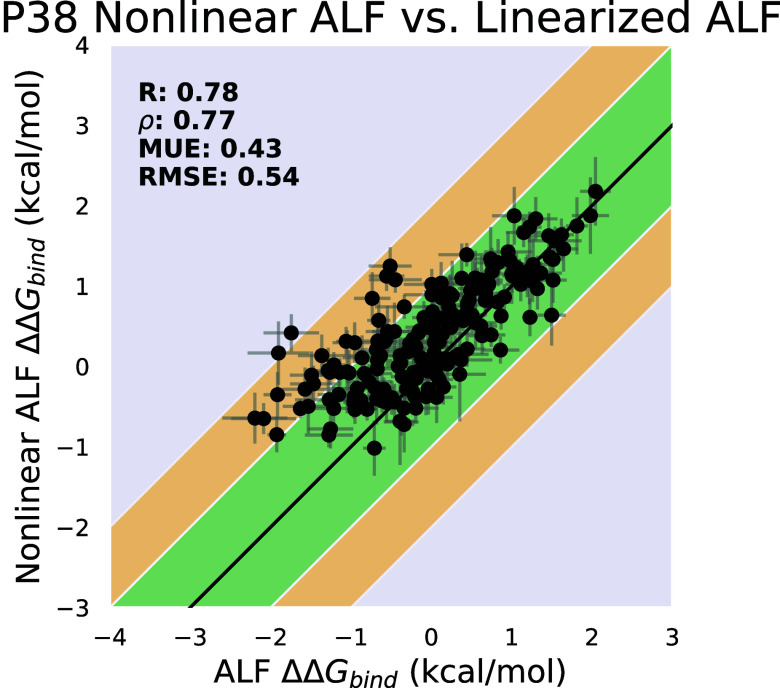
Correlation of ΔΔ*G*_bind_ values obtained with nonlinear vs linearized ALF protocols for the 192 combinatorial p38 ligands explored.

When looking at the ΔΔ*G*_bind_ values which result by subtracting the unbound Δ*G* values from those of the bound environment, the agreement between the results of both ALF protocols is slightly weaker (Figure 8). While there is a strong linear correlation between the free energies Δ*G* for both unbound and bound ligand environments, as shown in the previous paragraph, the Pearson R and Spearman ρ for the relative binding free energies ΔΔ*G*_bind_ fall to 0.775 ± 0.058 and 0.774 ± 0.068, respectively. This is largely due to the narrower range of values covered by ΔΔ*G*_bind_ than that of the ligand free energy Δ*G* in each environment. Consequently, the magnitude of the error remains relatively low, as quantified by the MUE and RMSE values of 0.429 ± 0.048 and 0.543 ± 0.057 kcal/mol, respectively. While this error is larger than initially expected, it falls within the statistical variability of the results, given practical limits of finite sampling and convergence. The variability for both ALF protocols is quantified by dividing trajectories into two groups that were processed independently and compared to each other. The deviation between halves was similar for both protocols, with MUE and RMSE values between halves of roughly 0.4 and 0.5 kcal/mol, respectively (see Supporting Information Figure S7). This suggests that comparable levels of statistical variation arise from both linearized and nonlinear ALF simulations and that with longer sampling, improved agreement between both protocols is expected.

Despite the moderate deviations between ALF protocols, both ALF protocols correlate well with experimental results. As shown in Table 6, the MUE and RMSE for both methods fall within 1 kcal/mol and show strong linearity and monotonicity (ranking capability) with experiment, as evidenced by the Pearson R and Spearman ρ coefficients, respectively.

**Table 6:** P38 System Comparison of λ Dynamics Results with Experiment

	subsetted system	full system
	linearized ALF	nonlinear ALF
R	0.753 ± 0.156	0.638 ± 0.229
Spearman ρ	0.735 ± 0.182	0.614 ± 0.271
MUE	0.545 ± 0.116	0.616 ± 0.147
RMSE	0.603 ± 0.125	0.713 ± 0.153

The lower accuracy of the full system results obtained with nonlinear ALF compared to those of the subsetted linearized ALF simulations is likely due to the larger chemical space involved. Nonlinear ALF and the independent bias on θ are improvements that made sampling the larger chemical space possible. Several factors may contribute to difficulty in sampling the larger chemical space, including poor bias potentials and slow degrees of freedom. First, as mentioned in the methods, for larger chemical spaces, some of the bias parameters converge to different values each time ALF is run, suggesting that the biases fit the higher dimensional alchemical landscapes less well. Improved bias functions are expected to improve sampling and convergence in high dimensional chemical spaces and will be the focus of future studies. Second, slow degrees of freedom that nonlinear ALF had less time to sample may have hindered convergence. The largest contributions to the difference arose from the bound calculations of subsets 2, 4, and 7, which contained some of the tightest bound ligands. These ligands also show some of the largest variability between the two halves of the linearized ALF results (Supporting Information Figure S7). Focusing more sampling on these tightly bound ligands with the screening methods described below in the discussion may improve accuracy.

Regardless of the lower accuracy observed for the nonlinear results, they require much less computational time with significantly less manual intervention. When comparing the ALF schedules for both protocols and the total simulation time needed to complete the sampling (as shown in Tables S2 and S3 for nonlinear and linearized ALF schedules, respectively), the single pair of nonlinear ALF simulations yielded converged results at 2.7 times less simulation time than the nine pairs of linearized ALF simulations. Notably, nonlinear ALF also needed much less manual intervention than did the linearized ALF approach, wherein one has to manually subset ligands and manipulate the input files accordingly. Thus, nonlinear ALF provides a more streamlined workflow for λ dynamics, allowing direct use of msld-py-prep output to examine a large series of ligand analogues. Furthermore, with a slight decrease in accuracy, at least in the case of p38, nonlinear ALF not only reduces potential for human error but also allows for a faster setup of the simulations, less simulation time, and greater exploration of chemical space.

## 4 Discussion

This work illustrates our development of a θ biasing potential and nonlinear ALF scheme for exploration of a significantly increased number of perturbations at a single site with λ dynamics. This new capability has significant implications in computational protein design and computer-aided drug design.

In protein design, there are 20 naturally occurring amino acids, and experimental design protocols often employ site-saturation mutagenesis to explore all substitutions at a single site. Previous simulations were limited to examining only a subset of these mutations at one time. For example, if all 20 amino acids were of interest, they could be grouped into three or more separate calculations with a shared reference sequence, but this required more user effort and more computational resources. This scaled poorly if interactions between *M* different interacting sites were important because 3^*M*^ calculations were required to capture all interactions between possible amino acids. Experimental studies of all possible mutations at four interacting sites are common,^64,65^ and larger numbers of sites would be of interest if they were accessible. Previous studies with λ dynamics have focused on making robust free energy predictions for large numbers of sites,^37,40^ and together with the present work, they enable exploration of all possible amino acids at each of those sites simultaneously.

In computer-aided drug design, different considerations apply. One can explore coupling between many substituents at multiple ligand perturbation sites more efficiently with the developments presented in this work. The p38 system described above previously required breaking the first site into 3 groups and the second site into 3 groups for 9 total simulations but can now be evaluated within a single simulation. However, medicinal chemists typically optimize a single site at a time, assume sites are largely independent, and are interested in more than 20 possible chemical substituents at each site. Consequently, the approaches described here are likely to be of greater interest for sampling 20 to 50 perturbations at a single site, rather than exploring coupling between sites.

The ability to evaluate many substituents within a single simulation makes λ dynamics significantly more efficient than conventional free energy methods like FEP and TI, which require roughly a dozen simulations for each pairwise comparison. While the sampling requirements of λ dynamics do increase somewhat for larger numbers of ligands, previous studies tend to find the largest efficiency gains over FEP and TI for systems with multiple perturbation sites because they include many more ligands.^5^ In this study, uncertainty levels differed but control simulations took significantly more computational effort per ligand than the many substituent simulations by a factor of 3.6 in 1,4-disubstituted benzene solvation, a factor of 4.2 in protein G stability, and a factor of 2.7 in p38 ligand binding. With the ability to sample many substituents at a single site demonstrated in this work, it is likely that the relative efficiency of single site λ dynamics simulations will also improve significantly. Benchmarking studies are needed in this area, as it is possible that a few poorly behaved ligands could distort the structure or lead to unbinding and compromise the accuracy of λ dynamics for the remaining well-behaved ligands.

Early λ dynamics studies frequently mentioned using λ dynamics in screening mode,^29,66,67^ analogous to a competitive binding assay, but this idea has not been explored further. In screening mode, ALF is used to flatten the alchemical landscape in the reference ensemble (the solvated ensemble for ligand binding or the unfolded ensemble for protein folding), which is often the less expensive ensemble to simulate. Simulations are then run in the other ensemble using the biases from the reference ensemble. Consequently, only the most favorable ligands or sequences are sampled, and sampling time is not wasted quantifying exactly how unfavorable bad ligands or sequences are. This idea is worth revisiting in future studies in light of the ability to sample much larger numbers of perturbations within a single calculation. Such a scheme would also discourage poorly behaved ligands from disrupting the binding site.

Finally, while these methods significantly increase the number of substituents which may be sampled at a single site, limitations still apply. In principle, the independent bias on θ can sample arbitrarily large numbers of substituents at a site due to the careful balancing between the free energy of the one dominant substituent and the remaining substituents in eq 12, (provided the implicit constraint *c* value is slowly increased to appropriate values to allow the dominant substituent to reach λ values of 0.99, e.g. 8.5 for 1000 substituents). In contrast, nonlinear ALF scales like *N*_*s*_^2^ because there are *O*(*N*_*s*_^2^) coefficients that must be optimized to independently flatten barriers from any of the *N*_*s*_ substituents to any of the other *N*_*s*_ – 1 substituents. This limits nonlinear ALF to roughly 50 to 100 substituents. Further developments to overcome this limit may be possible, as many bias coefficients are correlated,^34^ and will be the subject of future work.

## 5 Conclusions

λ dynamics is a highly efficient and scalable alchemical free energy method, but in practice, λ dynamics was limited to exploring 8–9 different substituents per site. In this work, we presented a bias on θ to significantly increase the fraction of time spent sampling physical states, and an adaptive landscape flattening algorithm with improved scaling, raising this limit to 50–100 substituents. The ability to look at large numbers of perturbations raises new possibilities for applying λ dynamics in computational protein design and computer-aided drug design.

## Data Availability

Example run input files and scripts used for landscape flattening are available for download at https://github.com/RyanLeeHayes/PublicationScripts/blob/main/2024ManySubs.tgz. ALF scripts are available at https://github.com/ryanleehayes/alf. Modifications to CHARMM, including the new θ biases, have been submitted to the developers version of CHARMM and will become available in a future release.

## Supporting Information Available

Alternative free energy cycles, discussion of free energy estimators, other considered biases, methodology for independent bias coefficient determination, visualization of improved sampling with the collective bias, details for linearized and nonlinear ALF, additional results for 1,4-disubstituted benzene, protein G, and p38, and details of uncertainty calculations . (PDF)
