# Supplementary material for: How to Sample Dozens of Substitutions per Site with λ Dynamics
Source: J Chem Theory Comput. 2024 Jul 8;20(14):6098–110. doi: 10.1021/acs.jctc.4c00514 (PMC11270746; doi:10.1021/acs.jctc.4c00514)
Supplement: Supplementary file 1 — ct4c00514_si_001.pdf [file ct4c00514_si_001.pdf]

Supporting Information for: How to Sample Dozens of Substitutions per Site with λ Dynamics

Ryan L. Hayes,[*,†,‡] Luis F. Cervantes,[¶] Justin Cruz Abad Santos,[†] Amirmasoud Samadi,[†] Jonah Z. Vilseck,[§,‖] and Charles L. Brooks III[*,⊥,#]

†*Department of Chemical and Biomolecular Engineering, University of California Irvine 92697*

‡*Department of Pharmaceutical Sciences, University of California Irvine 92697*

¶*Department of Medicinal Chemistry, College of Pharmacy, University of Michigan 48109*

§*Department of Biochemistry and Molecular Biology, Indiana University School of Medicine 46202*

‖*Center for Computational Biology and Bioinformatics, Indiana University School of Medicine 46202*

⊥*Department of Chemistry, University of Michigan 48109*

#*Biophysics Program, University of Michigan 48109*

E-mail: rhayes1@uci.edu; brookscl@umich.edu

S1 Free Energy Diagrams

Figure 1 in the main text describes how to compute a relative free energy of two ligands binding to the same protein. In this case the physical process transfers a ligand from the unbound, solvated ensemble to the protein bound ensemble and the alchemical process transforms from one ligand to another.

This manuscript also evaluates two other relative free energies: the relative solvation free energy of two ligands and the relative folding free energy of two proteins. For relative solvation free energies, the physical process transfers the ligand from the vacuum ensemble to the solvated ensemble, and the alchemical process transforms from one ligand to another. For relative folding free energies, the physical process transitions the protein from the unfolded ensemble to the folded ensemble, and the alchemical process transforms the protein from one sequence to another.

Many other relative free energies are possible. Relative free energies of protein-protein binding can be computed with a physical process that transitions between dissociated and bound ensembles, and an alchemical process that transforms from one sequence to another. Relative protein-ligand binding upon protein mutation uses a physical process that transitions the protein from the apo ensemble to the holo ensemble and an alchemical process that transforms from one sequence to another. Relative solvation free energies of ligands in different solvents are related to partition coefficients and involve a physical process that transfers a ligand from the ensemble of one solvent to the ensemble of the other, and an alchemical process that transforms from one ligand to another.

S2 Free Energy Estimation

Free energy estimation utilizes the histogram-based estimator described in the main text. This is a biased free energy estimator because with infinite sampling, it will not converge to

the exact free energy value for the force field. Instead it converges to a nearby value that includes contributions from other alchemical states with $\lambda > \lambda_c$. The alchemical trajectory is saved every 10 steps. While these snapshots are strongly correlated, numerical experiments have shown that even moderate decreases in the sampling rate result in larger errors in the free energy estimates. Conversely, moderate increases in the sampling rate result in excessively large files.

Other free energy estimators, such as the multistate Bennett acceptance ratio (MBAR)[1] or Rao-Blackwell estimator[2–4] can provide unbaised estimates, but are not practical for λ dynamics. The Rao-Blackwell estimator cannot be solved analytically for continuous λ dynamics with potentials that are nonlinear functions of λ, and many of the interactions in λ dynamics, such as soft-core interactions, are nonlinear functions of λ. The MBAR estimator can in principle be used, but would incur prohibitive computational overhead. The MBAR estimator requires evaluation of energies for all alchemical end states for every snapshot. Even for the small alchemical spaces considered here, computing 20 energies every 10 steps would triple the number of energy evaluations and the computational cost. Typically, though not always, only the snapshots near an alchemical end state contribute to the free energy, so MBAR is in some ways just an excessively expensive reweighting scheme that ensures fully rigorous free energy estimates. Subsampling to the point that MBAR becomes practical will likely degrade accuracy more than the systematic bias introduced by the histogram-based estimator.

Several features decrease the bias introduced by the histogram-based estimator. The first is the use of a narrow bin, with $\lambda_c = 0.99$. This prevents too many states with very different free energy from contributing. The next is the use of ALF, which approximately flattens endpoint traps, so states near the endpoint typically do not differ substantially in free energy. The use of soft-cores also makes the free energy profiles smoother near the endpoints, so the finite width contributes less error; indeed, soft cores are essential with λ dynamics precisely because hard cores introduce significant bias into the histogram-based estimator.[5] Finally, the use of implicit constraints focuses more sampling on states near $\lambda = 1$ and less on states near $\lambda = \lambda_c$, further reducing bias.

S3 Other Considered Biases

We also considered a cubic λ bias that penalized states with three or more non-zero λ values, but which does not affect the energy of transitions with only two non-zero λ values. The goal was to penalize alchemical intermediates without adding a barrier to direct transitions. For a three substituent system, this bias takes the form

$$U_{\text{Bias}} = \alpha\lambda_1\lambda_2\lambda_3 \tag{S1}$$

$$\lambda_1\lambda_2\lambda_3 = \sum_i^3 \frac{1}{3}\lambda_i(\lambda_i - 0.5)(\lambda_i - 1) \tag{S2}$$

By analogy, it can be shown that

$$U_{\text{Bias}} = \alpha \sum_i^{N_s} \frac{1}{3}\lambda_{si}(\lambda_{si} - 0.5)(\lambda_{si} - 1) \tag{S3}$$

sums over all unique triple products of lambda (e.g. $\lambda_1\lambda_2\lambda_3 + \lambda_1\lambda_2\lambda_4 + \lambda_1\lambda_3\lambda_4 + \lambda_2\lambda_3\lambda_4$ for $N_s = 4$), and effectively biases against any state with more than three non-zero λ values. Unfortunately, this approach was rapidly abandoned because unreasonably large values of the coefficients of several thousand kcal/mol were required to maintain high FPL.

A precursor to the independent bias described in the main text is given by

$$U_{\text{Bias}} = \sum_s^M \sum_i^{N_s} -b\log(N_s-1)\exp(-c(1+\sin(\theta_{si}))) \tag{S4}$$

where M is the number of sites, and the $\log(N_s - 1)$ scaling of the potential is designed to ensure that for $b \approx k_BT$, as the number of θ_i variables increases, on average only one or two of them can escape from the trap around $\theta = -\pi/2$ at a time. This bias is in some ways more ideal than the bias in the main text because it is slightly narrower, and maintains a better FPL relative to the collective bias, but

the form in the main text was chosen to more closely mimic the form of the collective bias. The coefficient $kT\log(N_s-1)$ in this function also fails to account for the narrowing of the well near $\theta = -\pi/2$ as it grows deeper, so the fraction physical ligand will continue to fall slightly as N_s grows.

Another unexplored approach is to tinker with the functional form of the implicit constraints, but this will be difficult to tune, and also means that if a favorable physical conformation for a particular ligand appears, it can't immediately pop out of the energetic trap that keeps λ low, but would have to diffuse through a large amount of alchemical space to get to a position where it could turn on. This is because modifying the implicit contraints penalizes states with many non-zero λ values entropically rather than energetically, so there will be large stretches of alchemical space where each λ value is supressed.

S4 Independent Bias Derivation

The bias coefficient of the independent bias in the main text may be derived as follows. The energy of the trap may be approximated as $-b+b(\theta-3\pi/2)^2$, which gives a free energy G_- of

$$G_- = -b - kT\ln\sqrt{\frac{2\pi kT}{b}} \tag{S5}$$

while the energy from 0 to π may be approximated as 0, and gives a free energy G_+ of

$$G_+ = -kT\ln\pi \tag{S6}$$

One can ensure that roughly two substituents out of N_s are in the state between 0 and π by ensuring the free energy G_- is $kT\ln((N_s-2)/2)$ lower than G_+. Replacing N_s-2 with N_s gives rise to the expression in the main text. To instead ensure on average only one substituent out of N_s is in the state between 0 and π, G_- should be $kT\ln(N_s-1)$ lower than G_+, giving rise to the alternative expression

$$\frac{b}{kT} = \frac{1}{2}\ln\left(\frac{\pi {N_s}^2}{2}\frac{b}{kT}\right) \tag{S7}$$

S5 Improvement in Sampling with Indepdendent-2 θ Bias

The functional form of the implicit constraints gives rise to an entropic term that helps focus sampling on the endpoints, and the Independent-2 bias further adds an enthalpic term to focus sampling on the endpoints. The 1,4-substituted benzene system described in the Main Text has $N_s = 24$ substituents, and Figure S1 demonstrates the improvements in sampling the Independent-2 bias gives in this system. The free energy of implicit constraint entropy and θ bias enthalpy is determined by Monte Carlo sampling. With no θ bias (top panel), no physical ligand with $\lambda_c > 0.99$ is sampled. However, the Independent-2 θ bias (middle panel), effectively samples both endpoints, with free energy wells near $\lambda = 0$ and $\lambda = 1$ revealing increased sampling of these states. The bottom panel shows the residual free energy profiles of 1,4-substituted benzene as a function of the various λ_{si} values at the first site after ALF has flattened the landscape. These residual free energy profiles have the free energy in the middle panel subtracted off so that ALF does not try to flatten the favorable sampling of the end states introduced by the implicit constraints and the θ bias. The total free energy in production simulations is thus the sum of the middle and bottom panels. In practice, evaluation of whether free energy profiles in the bottom panel appear flat is often used to determine whether ALF has converged.

S6 Adaptive Landscape Flattening Details

The biases used by ALF include the linear bias, the quadratic bias, the skew bias, and the end-

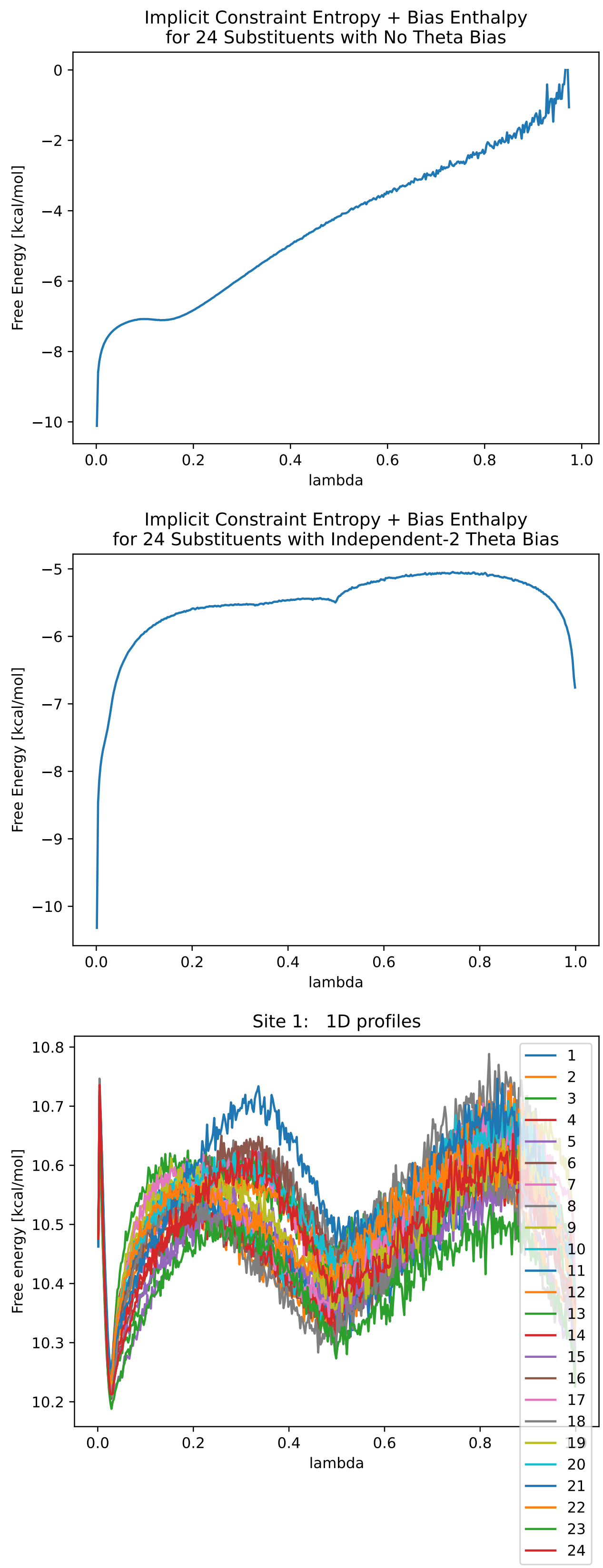

Figure S1: The free energy contributions of implicit constraint entropy and θ bias enthalpy for (top) no θ bias and (middle) the Independent-2 θ bias. (Bottom) the free energy profiles after flattening with ALF with the free energy contribution in the middle panel removed.

point bias. The linear bias is

$$U_\phi = \sum_s^M \sum_i^{N_s} \phi_{si}\lambda_{si} \tag{S8}$$

This bias is tuned to ensure all substituents have the same free energy and can be sampled in the same simulation. The quadratic bias is

$$U_\psi = \sum_s^M \sum_i^{N_s} \sum_{j=i+1}^{N_s} \phi_{si,sj}\lambda_{si}\lambda_{sj} + \sum_s^M \sum_{t=s+1}^M \sum_i^{N_s} \sum_j^{N_t} \phi_{si,tj}\lambda_{si}\lambda_{tj} \tag{S9}$$

The quadratic bias covers most of electrostatic interactions. The second term is optional and accounts for intersite coupling, and is not always used. In this work all intersite terms are omitted. The skew bias is

$$U_\chi = \sum_s^M \sum_i^{N_s} \sum_{j\neq i}^{N_s} \chi_{si,sj}(1-\exp(-\lambda_{si}/\sigma))\lambda_{sj} + \sum_s^M \sum_{t\neq s}^M \sum_i^{N_s} \sum_j^{N_t} \chi_{si,tj}(1-\exp(-\lambda_{si}/\sigma))\lambda_{tj} \tag{S10}$$

where $\sigma = 0.18$. The skew bias was originally unnecessary, but gave improved fits to free energy profiles once the use of soft cores became more common. The second term also accounts for intersite coupling, but even when used is tightly regularized because it is nearly degenerate with the intrasite terms. The endpoint bias is

$$U_\omega = \sum_s^M \sum_i^{N_s} \sum_{j\neq i}^{N_s} \omega_{si,sj}\frac{\lambda_{si}}{\lambda_{si}+a}\lambda_{sj} + \sum_s^M \sum_{t\neq s}^M \sum_i^{N_s} \sum_j^{N_t} \omega_{si,tj}\frac{\lambda_{si}}{\lambda_{si}+a}\lambda_{tj} \tag{S11}$$

where $a = 0.017$. The endpoint bias covers the cost of moving solvent and other environment atoms out of the way to make room for the excluded volume of a substituent before λ is large enough for other interactions to stabilize its presence. Like the skew bias, the second

term accounts for coupling between sites, but is tightly regularized because it is nearly degenerate with intrasite terms.

The flatness of the free energy landscape is assessed by binning samples into 3 or 4 kinds of histograms that are used to compute free energy profiles. There are N_s 1-D profiles that bin samples into B bins from 0 to 1 for each λ values. There are $N_s(N_s-1)/2$ transition profiles, which for $\lambda_{si}+\lambda_{sj} > 0.8$ bin $\lambda_{si}/(\lambda_{si}+\lambda_{sj})$ into B bins between 0 and 1; this measures the free energy along transitions directly from $\lambda_{sj}=1$ to $\lambda_{si}=1$. There are also $N_s(N_s-1)/2$ 2-D profiles, which divide λ_{si} and λ_{sj} into $\sqrt{B}$ bins from 0 to 1 in each direction; these profiles are only included for $N_s \geq 3$. These profiles are present for each site, if there are multiple sites, there will be more profiles. If desired, for multisite systems there are also N_sN_t intersite 2-D profiles for every combination of sites s and t divide λ_{si} and λ_{tj} into $\sqrt{B}$ bins from 0 to 1 in each direction.

These free energy profiles are determined by binning samples from frames of λ trajectories. Samples are typically taken from the last 5 cycles of ALF, and not just the last cycle, to provide a more comprehensive view of the free energy landscape. Since the last 5 cycles of ALF were each run with a different biasing potential, the samples are combined together with WHAM/MBAR. The total free energies of each simulation may be computed as

$$f_i = -\beta^{-1} \ln \sum_t^{\text{Frames}} \frac{\exp(-\beta U_i(\vec{\lambda}_t))}{\sum_k^K N_k \exp(\beta f_k - \beta U_k(\vec{\lambda}_t))} \tag{S12}$$

where f_i is the free energy of cycle i of ALF, $\beta = 1/(kT)$, U_i is the biasing potential from the ith cycle of ALF, $\vec{\lambda}_t$ are the alchemical coordinates from frame t drawn from any cycle, K is the number of included cycles, and N_i is the number of frames from the ith cycle. We note that within the ALF implementation, U_K for the most recent cycle of ALF is set to 0, and U_i of the remaining cycles is set to $U_i - U_K$. Once the free energies f_i are optimized by iteration, the weight of a particular frame in the potential of the final cycle K may be computed as

$$w_{Kt} = \frac{\exp(-\beta U_K(\vec{\lambda}_t))}{\sum_k^K N_k \exp(\beta f_k - \beta U_k(\vec{\lambda}_t))} \tag{S13}$$

where w_{Kt} is weight in potential K from frame t. These weights are then added to bins based on the associated alchemical coordinate, and the free energy of each bin is determined from

$$G_{pb} = -\beta^{-1} \ln \sum_t^{\text{Frames}} w_{Kt} u_{pb,t} \tag{S14}$$

where G_{pb} is the free energy of profile p, bin b, and $u_{pb,t}$ is an indicator function that is 1 if frame t is in profile p, bin b, and 0 otherwise.

In addition to estimating free energy profiles, estimates are required for how the free energy profile will change as the bias changes. For linearized ALF, the required quantity may be computed as

$$\frac{\partial G_{pb}}{\partial \alpha_i} = \frac{\sum_t^{\text{Frames}} \frac{\partial U_K(\vec{\lambda}_t)}{\partial \alpha_i} w_{Kt} u_{pb,t}}{\sum_t^{\text{Frames}} w_{Kt} u_{pb,t}} \tag{S15}$$

where $\partial G_{pb}/\partial \alpha_i$ is the change in free energy profile p, bin b, with respect to a change in biasing potential parameter α_i, where α may represent a ϕ, ψ, χ, or ω coefficient, and $\partial U_K(\vec{\lambda}_t)/\partial \alpha_i$ is the change in the bias potential for frame t, with respect to a change in α_i. For example, if α_i is ϕ_{si}, then $\partial U_K(\vec{\lambda}_t)/\partial \phi_{si}$ is $\lambda_{si,t}$, the λ_{si} value from frame t.

In the case of the linearized loss function

$$L_{\text{Linear}} = \sum_i^{\text{Biases}} k_i(\alpha_i - \alpha_{i,0})^2 + \sum_p^{\text{Profiles}} \sum_b^{\text{Bins}} k_{pb}(G_{pb} + \Delta G_{pb}(\vec{\alpha}) - G_{pb,\text{Imp}} - \bar{G}_p)^2 \tag{S16}$$

$$\Delta G_{pb}(\vec{\alpha}) = \sum_i^{\text{Biases}} \frac{\partial G_{pb}}{\partial \alpha_i} \Delta \alpha_i \tag{S17}$$

the loss function is quadratic, and may be minimized by setting the gradient with respect to $\vec{\alpha}$ equal to zero and solving the linear system of equations through matrix inversion.

For nonlinear ALF, the required quantity is

$$G_{pb}(\vec{\alpha}) = -\beta^{-1} \ln \sum_{t}^{\text{Frames}} w_{\vec{\alpha}t} u_{pb,t} \quad \text{(S18)}$$

where $G_{pb}(\vec{\alpha})$ is the predicted free energy of profile p, bin b, for biasing coefficients $\vec{\alpha}$, and

$$w_{\vec{\alpha}t} = \exp(-\beta(U_{\vec{\alpha}}(\vec{\lambda}_t) - U_K(\vec{\lambda}_t)) w_{Kt} \quad \text{(S19)}$$

where $U_{\vec{\alpha}}(\vec{\lambda}_t)$ is the bias energy of frame t with the proposed bias coefficients $\vec{\alpha}$. Derivatives of $G_{pb}(\vec{\alpha})$ with respect to α_i are required for nonlinear ALF optimization, and are tedious to write, but straightforward to take using the chain rule.

The coefficients k_{pb} and k_i are tuned to achieve an appropriate balance between flattening, log likelihood, and regularization, and to remove B dependence of the loss function. The number of bins needs to be a square for the 2D profiles, and for nonlinear ALF needs to be a multiple of 32 (the number of threads in a CUDA warp) to simplify hardware optimization. A larger value of $B = 400$ was needed for linearized ALF, but due to likelihood optimization, nonlinear ALF requires fewer bins ($B = 64$ or $B = 256$) to identify and flatten trapped degrees of freedom.

For linearized ALF, the k_{pb} coefficient is 1 for 1D profiles, $2/(N_s - 1)$ for transition and 2D profiles, and $1/(N_s N_t)$ for intersite 2D profiles between sites s and t, and is $B/4 = 100$ times larger for the last bin in 1D and 2D intersite profiles

The regularization constant k_i is $1/2^2$ for linear bias coefficients ϕ, $1/8^2$ for quadratic bias coefficients ψ, $1/2^2$ for skew bias coefficients χ, $1/1^2$ for endpoint bias coefficients ω, $1/2^2$ for intersite ψ terms, and $1/0.5^2$ for intersite χ and ω terms, where α_i is an arbitrary biasing coefficient encompassing any ϕ, ψ, χ, or ω coefficients, and $\alpha_{i,0}$ is the value the bias is regularized to, which for most biases will be the previous value of that coefficient so that $\alpha_i - \alpha_{i,0} = \Delta\alpha_i$, but for intersite χ and ω terms is 0 so that $\alpha_i - \alpha_{i,0} = \alpha_i$. Changes in bias coefficients are capped at $\frac{3}{2}k_i^{-1/2}$; if any bias coefficient changes by more than this amount, all bias changes are scaled so that no change is greater than the cap.

For nonlinear ALF, the k_{pb} terms are the same as in the linearized case, but scaled by $1/(B(kT)^2)$ to make the loss function dimensionless and ensure the first term of $L_{\text{Nonlinear}}$ is roughly independent of the number of bins. The k_i terms were scaled by $1/(400(kT)^2)$ to ensure their relative strength remains the same as it was in the linearized loss function with $B = 400$.

Convergence of ALF runs was monitored with various ALF routines in the folded ensemble of the 22 substituent protein G test system. A reference run was performed using nonlinear ALF, $B = 256$ with likelihood maximization, to determine an independent estimate of the biases. The reference run included 300 flattening rounds of 100 ps, 30 flattening rounds of 1 ns, 5×5 ns production, 5×20 ns production, and 5×100 ns production. Biases at the end of this reference calculation were compared to biases using shorter independent flattening runs. Final biases in several cases including the reference trajectory show ALF has blocked a few transition paths, often raising the ψ bias by a couple hundred kcal/mol, and shifting the ω biases by 5-10 kcal/mol. The nonunique bias parameters are largely responsible for the fact that the root mean square bias deviations for the quadratic bias in Figure S2 do not converge to 0. Similar plots apply for the skew and endpoint biases.

Nonlinear ALF is halted when the root mean square change in biasing parameters falls below a certain threshold two times in a row. A threshold of 2.5×10^{-3} kcal/mol was found to converge in a comparable number of ALF cycles to smaller thresholds, while larger thresholds failed to converge. We chose a slightly smaller threshold of 1.25×10^{-3} kcal/mol because we determined this brought the root mean square difference of the bias within 0.01 kcal/mol of the final value obtained with much smaller thresholds, and ALF has historically truncated the bias parameters at the hundredths place.

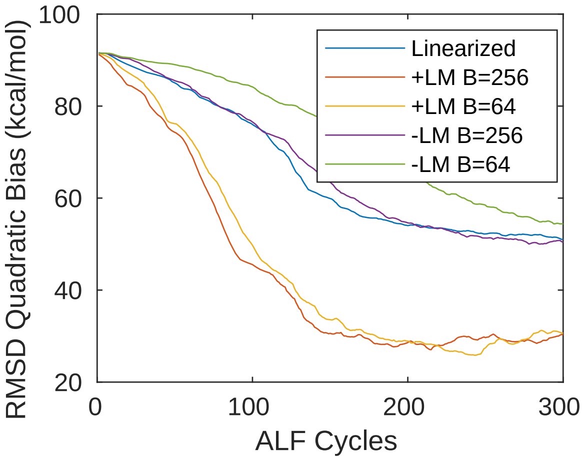

Figure S2: The convergence of the quadratic bias parameters as a function of ALF cycles for the folded ensemble of the 22 substituent Protein G test system. Convergence is quantified by the root mean square difference (RMSD) of all bias parameters relative to final biases obtained from an independent ALF run after long 5×100 ns production runs, using nonlinear ALF with likelihood optimization and $B = 256$. Nonlinear ALF with likelihood optimization (+LM) and with 256 bins provides the fastest convergence.

S7 Additional 1,4-substituted Benzene Results

Slightly larger errors are observed for the 24×24 system than for the 8×8 system because the larger system has 9 times more ligands to sample, but only 2.5 times more sampling. Intermediate errors are observed in comparing the 8×8 and 24×24 systems because only one of the free energy values rather than both are drawn from the noisier 24×24 estimates. Solvation free energy estimates are noisier because they include error from the rapidly converging vacuum ensemble and the more slowly converging solvated ensemble.

The differences in main text Table 3 are roughly consistent with computational uncertainties in Table S1. The uncertainties in Table S1 apply to both molecules for each pair of molecules in main text Table 3, thus the uncertainty of the vacuum 8×8 calculations of 0.030 kcal/mol, gives rise to an expected RMSE of $\sqrt{0.030^2 + 0.030^2} = 0.042$, (the uncertainties are uncorrelated so they may be combined by a pythagorean sum), which is close to the observed value of 0.053 kcal/mol in main text Table 3.

Table S1: Root Mean Square Unncertainty of Calculated 1,4-substituted Benzene Solvation Free Energies

	Vacuum	Solvent	Solvation
8×8	0.030	0.048	0.060
24×24	0.075	0.112	0.138

The 8×8 system shows similar consistency with experimental measurements to the 24×24 systems shown in the main text (Figure S2).

S8 Additional Protein G Results

The results for protein G run with fswitch electrostatics (with a 10 Å switching radius and 12 Å cutoff) are shown in Figure S4. These results are similar to the PME results shown in the main text, but of slightly lower quality

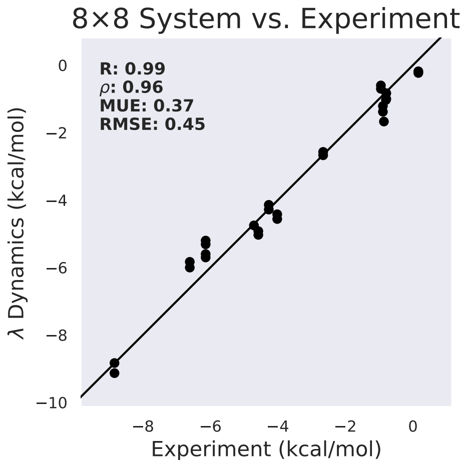

Figure S3: Correlation between λ dynamics calculations and experimental results for solvation free energies of 1,4-substituted benzene derivatives in the 8×8 system setup. RMSE and Pearson correlation values of 0.450 ± 0.102 kcal/mol and 0.987 ± 0.007 were obtained. The regions between ± 1 kcal/mol and ± 2kcal/mol are shaded in green and orange, respectively. The solid black line is $y = x$. Experimental data are cited in the main text.

when comparing their RMSE with experiment (main text Table 5). Surprisingly, the results are nearly on par with the PME results if the PME discrete solvent correction is applied to the fswitch results; however, there is no theoretical basis for adding this correction to fswitch results.

Since the experimental pH is not known, we plot the stability of the mutation T16H as a function of pH (Figure S5). The mutational stability difference is relatively constant above pH 7, given the unfolded state titrates around a pKa of 6.5. Below this pH, the destabilization by the histidine mutation rises steadily until the folded state also titrates near a pH of 3, and then levels out.

S9 Additional p38 Results

Single ensemble free energy results were obtained in both the water environment ensemble and the bound environment ensemble shown in the thermodynamic cycle in main text Figure 1. The single ensemble free energy results for the

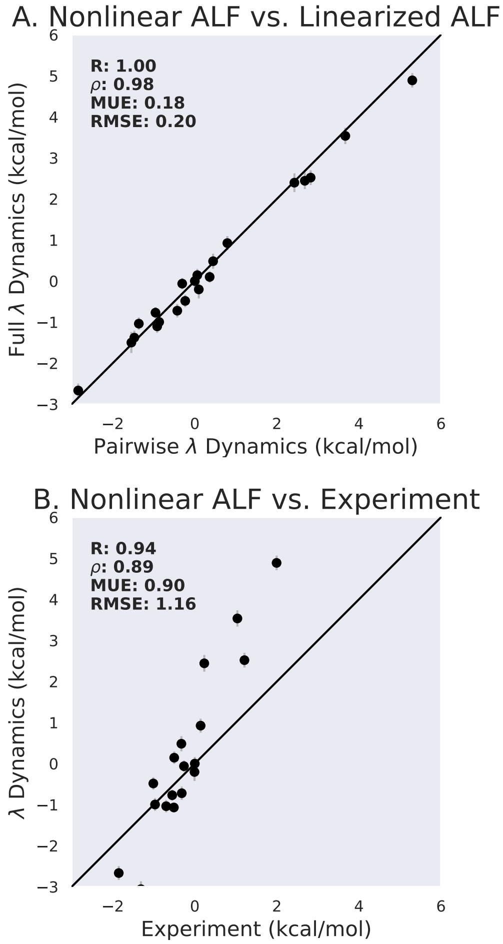

Figure S4: (A) 21 pairwise simulations (x-axis) and a single 22 substituent simulation (y-axis) run with fswitch electrostatics show excellent agreement in predicted folding free energies. (B) The single 22 substituent simulation also agrees reasonably with experiment with an RMSE of 1.165 ± 0.376 kcal/mol and a Pearson correlation of 0.943 ± 0.064. Experimental values are cited in the main text. The regions between ± 1 kcal/mol and ± 2kcal/mol are shaded in green and orange, respectively. The solid black line is $y = x$.

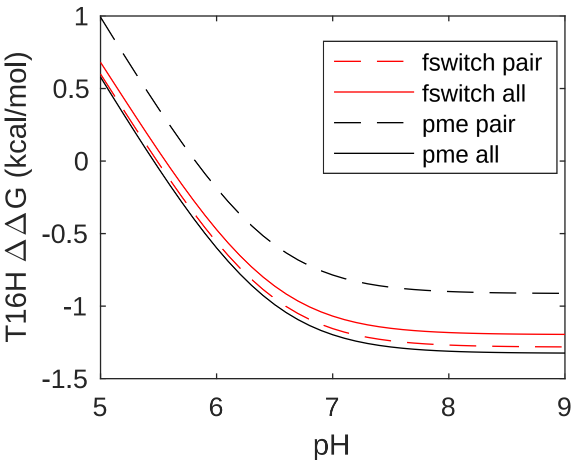

Figure S5: Predictions by λ dynamics simulations for the $\Delta\Delta G_{\text{fold}}$ of the T16H mutation, as a function of pH. Red curves use fswitch electrostatics, black curves use PME electrostatics. Dashed curves are calculated from the pairwise linearized ALF simulations, solid curves are calculated from the full system nonlinear ALF simulations. Between a pH of 6 and 8 the free energy predictions show relatively little variation.

total 192 combinatorial ligands sampled from each simulation set up are shown in Fig. S6. The relative binding free energies from these simulations are determined by subtracting the corresponding water free energy from the protein free energy calculated for each ligand compared to the reference.

In order to determine the expected deviation between the results of nonlinearized and linear ALF protocols due to finite sampling, a control test was conducted. This control involved splitting the simulations for each of the protocols into two groups, postprocessing each protocol's groups independently from one another, and comparing to the other group. For the nonlinearized protocol, a total of 15 trajectories were split into two groups of 7 full trajectories and half of the 15th trajectory. For the linearized protocol, for each subset of ligands (as shown in Table 6 of the main text), a total of 5 trajectories were split into two groups of 2 full trajectories and half of the 5th trajectory. This ensured that no time dependence effects were observed when comparing the results. Additionally, the same initial 5 ns of simulation for each trajec-

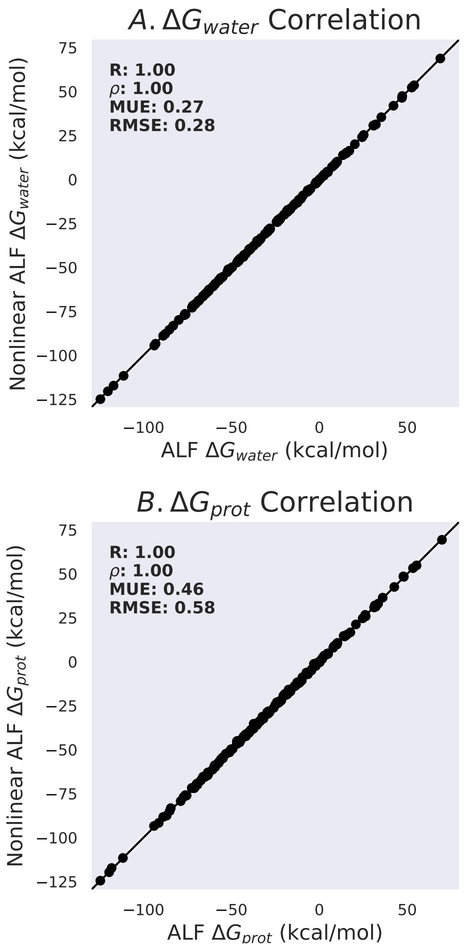

Figure S6: (A) Comparison between nonlinear and linearized ALF raw results for ligand-in-water simulations. (B) Comparison between nonlinear and linearized ALF raw results for bound ligand-in-protein simulations. The solid black line is $y = x$.

tory were discarded to allow for equilibration and also to remain as consistent with the original analysis shown in Fig. 8 of the main text.

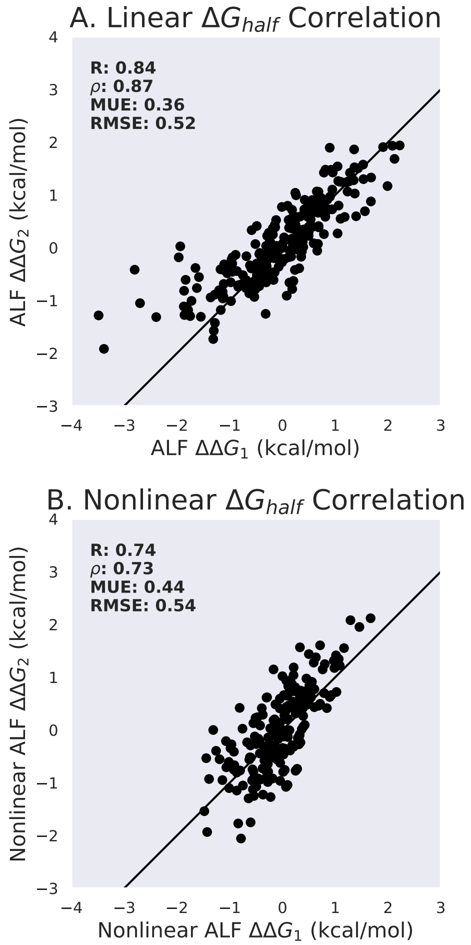

Figure S7: (A) Expected correlation between first and second halves of total trajectories run using the linearized ALF protocol for the total 192 combinatorial ligands sampled. (B) Expected correlation between first and second halves of total trajectories run using the nonlinear ALF protocol for the total 192 combinatorial ligands sampled. The regions between ± 1 kcal/mol and ± 2kcal/mol are shaded in green and orange, respectively. The solid black line is $y = x$.

S10 Uncertainty Calculations

An uncertainty value was calculated for the statistical metrics on the main text via the bootstrapping method called first-order normalized approximation.[6] For each particular comparison and for each trial in the bootstrapping process, a total of n samples (1,4-substituted benzene and protein G) or $n-1$ (p38) samples were selected randomly with replacement. The statistical metric (e.g. Pearson R, RMSE, etc.) was then calculated. This was repeated for a total of 1000 iterations. The reported statistic is computed from the original data (1,4-substituted benzene and protein G) or from the mean of the resulting distribution of 1000 samples of the metric (p38 binders). The reported uncertainty for the statistic is the distance between the mean of this distribution and the extreme of the symmetric 95% confidence interval.

Table S2: Linearized ALF splitting protocol for 9 different subsets of simulations required to span 192 combinatorial ligands sampled via nonlinear ALF for the p38 ligand system. The fragments included in groups A-C per site are shown in the main text Figure 7.

Subset	Site1	Site2
1	A	A
2	B	B
3	C	C
4	A	B
5	A	C
6	B	A
7	B	C
8	C	A
9	C	B

References

(1) Shirts, M. R.; Chodera, J. D. Statistically Optimal Analysis of Samples from Multiple Equilibrium States. *Journal of Chemical Physics* **2008**, *129*, 124105.

(2) Ding, X.; Vilseck, J. Z.; Hayes, R. L.; Brooks, C. L., III Gibbs Sampler-Based λ-Dynamics and Rao-Blackwell Estimator for Alchemical Free Energy Calculation. *Journal of Chemical Theory and Computation* **2017**, *13*, 2501–2510.

(3) Vilseck, J. Z.; Ding, X.; Hayes, R. L.;

Table S3: Nonlinear ALF Schedule and Total Simulation Time

Environment	Schedule	Total Simulation Time (ns)
Protein	200 100 ps iterations	2
	20 1 ns iterations	20
	15×25 ns replicas	375
	15×95 ns replicas	1425
Water	200 100 ps iterations	2
	20×1 ns iterations	20
	15×5 ns replicas	75
	15×25 ns replicas	375
	TOTAL	2294

Table S4: Linearized ALF Schedule per p38 Simulation Subset and Total Simulation Time

Environment	Subset Index	Schedule	Total Simulation Time (ns)
Protein	1-9	200 100 ps iterations	18
	1-9	20 1 ns iterations	180
	1-9	5×5 ns replicas	225
	1-7,9	5×25 ns replicas	1000
	1-7,9	5×75 ns replicas	3000
	8	5×50 ns	250
Water	1-9	200 100 ps iterations	18
	1-9	20 1 ns iterations	180
	1-9	5×5 ns replicas	225
	1,3, 5-9	5×20 ns replicas	700
	2,4	5×50 ns replicas	500
		TOTAL	6296

Brooks, C. L., III Generalizing the Discrete Gibbs Sampler-based λ-Dynamics Approach for Multisite Sampling of Many Ligands. *Journal of Chemical Theory and Computation* **2021**, *17*, 3895–3907.

(4) Robo, M. T.; Hayes, R. L.; Ding, X.; Pulawski, B.; Vilseck, J. Z. Fast Free Energy Estimates from λ-Dynamics with Bias-Updated Gibbs Sampling. *Nature Communications* **2023**, *14*, 8515.

(5) Hayes, R. L.; Armacost, K. A.; Vilseck, J. Z.; Brooks, C. L., III Adaptive Landscape Flattening Accelerates Sampling of Alchemical Space in Multisite λ Dynamics. *Journal of Physical Chemistry B* **2017**, *121*, 3626–3635.

(6) Puth, M.-T.; Neuhäuser, M.; Ruxton, G. D. On the Variety of Methods for Calculating Confidence Intervals by Bootstrapping. *Journal of Animal Ecology* **2015**, *84*, 892–897.
